# Polish Cystic Fibrosis Patients’ Health-Related Quality of Life and Its Influencing Factors: A Cross-Sectional, Single-Centre Study

**DOI:** 10.3390/healthcare12121183

**Published:** 2024-06-12

**Authors:** Magdalena Humaj-Grysztar, Marta Rachel, Joanna Bonior

**Affiliations:** 1 Institute of Nursing and Midwifery, Faculty of Health Sciences, Jagiellonian University Medical College, 31-501 Krakow, Poland; magdalena.humaj@uj.edu.pl; 2 Institute of Medical Sciences, Medical College of Rzeszow University, 35-310 Rzeszow, Poland; rachel@popia.pl; 3 Allergology Outpatient Department, Provincial Hospital No 2, 35-301 Rzeszow, Poland; 4 Department of Medical Physiology, Chair of Biomedical Sciences, Institute of Physiotherapy, Faculty of Health Sciences, Jagiellonian University Medical College, 31-126 Krakow, Poland

**Keywords:** cystic fibrosis, health-related quality of life, CFQ-R, lung function, exacerbation

## Abstract

Cystic fibrosis (CF) is a disease characterized by long-term and troublesome symptoms that affect the patient’s life. This study aimed to assess and compare the health-related quality of life (HRQoL) of Polish CF patients and identify factors influencing it. The study group consisted of 79 patients (6 to 42 years old), who filled in an age-appropriate Cystic Fibrosis Questionnaire-Revised. Medical data were collected from each patient’s medical records. The domains with the highest HRQoL median were eating problems (88.89), digestive symptoms (77.78) and physical functioning (75.00). The lowest-rated domain was social functioning (61.90). Age negatively correlated with eight domains, and most strongly with treatment burden (rho = −0.474). Physical functioning positively correlated with all spirometry parameters, and most strongly with FEV1% (rho = 0.588). Treatment burden, body image and respiratory symptoms were positively correlated with all spirometry parameters except PEF%. Present exacerbations reduced scores in almost all domains, and in the MANCOVA model they were a significant factor differentiating patients’ HRQoL. The univariate analysis of MANCOVA showed the significant effects of both health condition (F = 8.32, *p* = 0.005) and the COVID-19 pandemic (F = 5.89, *p* = 0.018) on social functioning domain, and of the place of residence on body image (F = 5.60, *p* = 0.21). A decreasing HRQoL with increasing age and during exacerbations indicates that it is important to focus on these aspects of patients’ lives and ensure they received the necessary support from their healthcare providers.

## 1. Introduction

### 1.1. Cystic Fibrosis

Cystic fibrosis (CF) is a rare, lifelong, genetically determined disease that is associated with shortened life expectancy and frequent exacerbations [1]. According to the European Cystic Fibrosis Society Patient Registry, in 2021 there were 54,043 CF patients on the registry, of which 1430 were Polish. The mean age of a CF patient in Europe, Israel and the Russian Federation was 22.3 years old and in Poland, 15.6 years old. A total of 55% of the people with CF living in Poland were female, and the majority of patients were less than 18 years old (64.6%) [2]. Due to the nationwide newborn screening that has been performed since 1 January 2009, the average age of CF diagnosis decreased, and in 2021 the median age at diagnosis was less than 3 months [2,3]. National Health Fund data show that in 2018 the average life expectancy of Polish CF patients was 24.5 years, while according to the Statistics Poland data, the average life expectancy in Poland was 81.8 years for females and 74.1 for males [4,5]. That difference is substantial, and it is a good example of the consequences associated with this disease.

Throughout the course of the disease, subsequent systems are gradually damaged, from the respiratory to the digestive system [1]. Fertility is also impaired, but cases of pregnancies and the birth of healthy babies to women with CF are known worldwide [1,6]. Due to the new therapies, and with cystic fibrosis transmembrane conductance regulator modulators, patients’ life expectancy and health-related quality of life (HRQoL) have increased [7,8]. Although the new drugs alleviate disease symptoms and improve everyday functioning, they are not available for everyone and are not free from side effects. Studies show that for some patients they can be associated with a deterioration in the quality of life and increased anxiety and depression [9,10].

Because CF is a systemic disease, patients require an interdisciplinary approach to their care, which can be challenging for healthcare professionals. To provide the best care, interdisciplinary medical teams should be established. In addition to doctors and nurses, these teams should also include psychologists, physiotherapists, nutritionists, social workers and pharmacists. Such teams, although not always covering all of the previously mentioned professionals, are working in cystic fibrosis treatment centres [11]. People with CF in Poland can be treated with CFTR modulators only at those centres where the “Treatment of patients with cystic fibrosis” drug program is implemented, and this covers the therapies with ivacaftor, ivacaftor with tezacaftor, ivacaftor with lumacaftor or ivacaftor with lumacaftor and elexacaftor. Patients who meet the conditions for inclusion in the drug program receive medications free of charge. Another drug program available for CF patients is “Treatment of chronic lung infections in beneficiaries with cystic fibrosis” [3]. There are currently 19 centres in Poland, and the largest one, in the Podkarpackie Voivodeship, is in the hospital where our study was carried out.

### 1.2. Quality of Life and Health-Related Quality of Life

The World Health Organisation defines quality of life as “an individual’s perception of their position in life in the context of the culture and value systems in which they live and in relation to their goals, expectations, standards and concerns” [12]. This definition suggests that QoL is a multi-aspect concept. It concerns aspects such as material comforts, relationships, personal safety, health and more. To assess how a specific disease, its’ treatment or health in general influences ones QoL, the health-related quality of life should be evaluated.

According to The International Society for Quality of Life Research’s Dictionary of Quality of Life and Health Outcomes Measurement, health-related quality of life is “A term referring to the health aspects of quality of life, generally considered to reflect the impact of disease and treatment on disability and daily functioning; it has also been considered to reflect the impact of perceived health on an individual’s ability to live a fulfilling life. However, more specifically HRQoL is a measure of the value assigned to duration of life as modified by impairments, functional states, perceptions and opportunities, as influenced by disease, injury, treatment and policy” [13]. Apart from the above definition, there are more. In chronic illnesses such as CF, it is crucial to monitor patients’ HRQoL, thus, specific questionnaires were developed.

### 1.3. Cystic Fibrosis and Health-Related Quality of Life Questionnaires

There are several tools for the health-related quality of life assessment of people living with CF. Some of the most frequently used are Cystic Fibrosis Quality of Life (CFQoL), Questions on Life Satisfaction (FLZ^M^-CF) and the Chronic Respiratory Disease Questionnaire (CRQ). Despite the wide range of available questionnaires, the Cystic Fibrosis Questionnaire-Revised (CFQ-R) is the most commonly used validated, disease-specific tool for assessing health-related quality of life for people with CF [14].

The Cystic Fibrosis Questionnaire (CFQ) was developed in France by Bernadette Henry et al. [15]. Nine domains were established: five general and four CF specific. Originally, the new tool was meant to be used by patients 14 years old and older (CFQ-14+), children aged 8 to 13 years old (CFQ-Child) and their parents (CFQ-Child P) [16]. Two versions of this specific tool for younger patients were separated in 2003—CFQ-R 6–11 and CFQ-R 12–13 [17].

A Polish adaptation of all four CFQ-R questionnaires was created based on their versions from the United States of America as part of the study COL/DPI/02/06. They were validated and published in 2009 by Sands and Borawska-Kowalczyk [18].

Frequent exacerbations, hospitalisations, the need to spend time on often time-consuming therapies and limitations in everyday functioning are some of the factors that can negatively affect patients’ quality of life. According to studies and systematic reviews, the most important factors are FEV1% (percent predicted forced expiratory volume in 1 second), patients’ sex, body mass index (BMI), age and pulmonary exacerbations [19,20,21,22].

Knowing the evidence from the literature regarding the impact of the previously mentioned factors affecting the quality of life of patients with CF, and, according to the authors’ knowledge, the lack of studies covering a wide age range in the population of Polish patients with CF, we aimed to assess and compare the health-related quality of life status of patients with CF in different age groups and to identify factors influencing it.

## 2. Materials and Methods

### 2.1. Study Design

This cross-sectional study included a population of CF patients of wide age range. It was conducted in accordance with the Helsinki Declaration and obtained acceptance from the Bioethics Committee of the Jagiellonian University Medical College, approval number 1072.6120.191.2018.

### 2.2. Setting

The data were collected in 2019 and 2020 at the Department of Allergology and Cystic Fibrosis and the Cystic Fibrosis Outpatient Clinic operating in the Provincial Clinical Hospital no. 2 in Rzeszow, Poland.

### 2.3. Participants

The study group consisted of convenience-sampled CF patients recruited during their visits to the hospital and outpatient clinic. Inclusion criteria were CF diagnosis, age 6–45 years old and our obtaining informed consent from the patient or his/her parent or legal guardian to participate in the study. Exclusion criteria were lack of CF diagnosis, age younger than 6 years old or over 45 years old, the presence of autoimmune diseases and refusal to participate in the study. Patients that met the inclusion criteria were approached by a member of the research team and informed about the aim and objectives of the study. They were also assured about the anonymity of the study and were informed that they could withdraw their consent at any time without having to give a reason. In the case of underage patients, all previously described information was also given to their parents or legal guardians. After obtaining the informed consent, they were asked to fill in the questionnaires.

### 2.4. Variables

The study took into account the following variables: health-related quality of life, spirometry results, patients’ medical data and sociodemographic characteristics.

Respondents were also assigned to one of two groups: patients in a stable health condition and those with present exacerbations. The inclusion criteria for exacerbations were onset or worsening of respiratory symptoms, weight loss, fever, malaise, worsening of FEV1% by at least 10% or physicians’ opinion that oral or intravenous antibiotic therapy was necessary.

Due to the COVID-19 pandemic outbreak and lockdown, during the data collection period our surveyed sample were also assigned into two groups according to their time of study participation: before the COVID-19 pandemic and during the COVID-19 pandemic.

### 2.5. Measurement

After our obtaining consent from each participant, they were asked to fill in the study questionnaire—the appropriate one for their age—Cystic Fibrosis Questionnaire-Revised. After that, a member of the research team collected their medical data from their medical records.

#### 2.5.1. Cystic Fibrosis Questionnaire-Revised

Questionnaires consisted of questions considering the socio-economic data of the respondents such as age, place of residence or marital status. To assess participants’ health-related quality of life, Cystic Fibrosis Questionnaire-Revised fitted for every age group: CFQ-R 6–11, CFQ-R 12–13 and CFQ-R14+ was applied [17].

Cystic Fibrosis Questionnaire-Revised (CFQ-R) is a validated tool created to assess CF patients’ quality of life. Depending on the respondents’ age, a specific questionnaire should be used. There are 3 versions designed for each age group: CFQ-R 6–11 for children from 6 to 11 years old, CFQ-R 12–13 for teenagers aged 12–13 and CFQ-R 14+ for teenagers aged 14 and older patients. CFQ-R14+ consists of 50 questions that are divided into 9 domains (physical functioning, role functioning, vitality, emotional functioning, social functioning, body image, eating problems, treatment burden and health perceptions) and 3 symptoms scales (weight, respiratory symptoms and digestive symptoms). Questionnaires for people younger than 14 years do not carry questions covering the domains of vitality, health perceptions, role functioning and weight. Every question in the questionnaire can be considered as negative or positive. Participants can answer questions by selecting one of the 4 answers based on a 4-point Likert scale. Results are calculated for each of the domains. The range varies from 0 points, which indicates the worst, to 100 points, which indicates the highest quality of life. For the purpose of the study, a Polish version of the questionnaires was used [17].

#### 2.5.2. Medical Data and Sociodemographic Characteristics

Parameters such as weight, height, body mass index (BMI), heart rate and saturation were collected from the patient’s medical records and used as independent variables. Also, spirometry results such as vital capacity (VC), forced vital capacity (FVC), forced expiratory volume in 1 second (FEV1), FEV1/FVC ratio (FEV1/FVC), peak expiratory flow (PEF), maximal expiratory flow (MEF 25, 50 and 75), and mid-maximal expiratory flow (MMEF) were collected. For the purpose of the study and statistical analysis, results were collected in the of calculated percent predicted values in accordance with patients’ height, weight, age, sex and racial background. Spirometry was performed in accordance with the recommendations of the European Respiratory Society and the American Thoracic Society [22,23].

Sociodemographic characteristics such as sex, age, place of residence, educational level and marital status were collected from patient’s medical data or from completed CFQ-R questionnaires.

### 2.6. Bias

Authors are aware of the bias that might be associated with convenience sampling; however, the number of possible participants was limited by the inclusion criteria and the number of potential participants. A member of the research team who collected the surveys offered each patient the opportunity to participate in the study, and patients decided whether they were interested in participating.

### 2.7. Study Size

Due to small sample size of CF patients living in the Podkarpackie Voivodeship, authors did not formally calculate a sample size and the study group consisted of convenience-sampled patients that at the time of data collection met the inclusion criteria and agreed to participate in the research. The final number of participants was 79 CF patients.

### 2.8. Quantitative Variables

Respondents’ quality of life results were described as a percentage, mean, median, interquartile range, minimum and maximum. Participants parameters such as age, weight, height, body mass index (BMI), heart rate, saturation and spirometry results were used as independent variables, therefore, they were not described and characterized in the study in the same way as the patients’ quality of life results.

### 2.9. Statistical Methods

To decide whether the parametric or non-parametric test would be used, an assessment of the quality of life data distribution was performed with the Shapiro–Wilk test (due to the relatively small study sample). Our data did not present normal distribution in all domains except for vitality (*p* = 0.56) and social functioning (*p* = 0.25), thus, non-parametric tests were used for analysis. For quantitative variables such as weight, age or spirometry results, Spearman’s correlation was performed to assess the correlation between those variables and the quality of life. The correlations were categorized in accordance with their strengths as follows [24]:

- rho ≥ 0.8, Very strong;
- rho = 0.7–0.6, Moderately strong;
- rho = 0.5–0.3, Fair;
- rho < 0.3, Poor.

To check if patients’ quality of life differs based on qualitative variables such as sex, place of residence, health condition or the presence of exacerbations and COVID-19 pandemic, Mann–Whitney U test was used. Due to the large difference in number of participants in the groups for the “place of residence” variable, it was decided to combine participants living in cities into one group for the purposes of statistical analysis. Therefore, after modifications, this variable, like the others, had only two categories.

To assess the association between quality of life domains, and categorical variables, multivariate analyses of covariance (MANCOVA) was performed. After the Box–Cox transformation of data to make its distribution closer to normal, and selection of the variables, a MANCOVA model was built. Model included physical functioning, social functioning, body image and respiratory symptoms as dependent variables, patients’ health condition (stable–exacerbations), place of residence (village–city) and COVID-19 pandemic (before pandemic–during pandemic) as categorical predictors and patients’ FEV% and FEV1% as covariates. Mardia’s multivariate skewness and kurtosis test was performed to check if our dependant variables met the multivariate normality assumptions. As *p* value for both skewness and kurtosis was larger than α = 0.05, we retained the null hypothesis and considered multivariate normality of the dependent variables. Additional Box M test was performed and confirmed the homogeneity of the variance–covariance matrices.

The significance level for the study was set at α = 0.05 and additional marginally significant level at 0.06 > α > 0.05. Data were analysed using the Statistica 13.3 software.

## 3. Results

### 3.1. Baseline Characteristics

The study consisted of 79 participants with CF. Most of them were females (59.5%). The vast majority of patients were 14 years old or older (78.5%). Adults accounted for 68.4% of the study group. The number of boys and girls (participants up to 17 years old) was almost identical (13 vs. 12). The difference between the group of men and women was more visible (19 vs. 35). More than ¾ of the adults were single (81.5%). The youngest patient was 6 years old and the oldest was 42 years old. The village was the most common place of residence (69.6%). Only 7.6% of patients declared that they have graduated from university. Most patients participated in the study during the exacerbation of their underlying disease (59.5%) and after the COVID-19 pandemic outbreak and lockdown in Poland (53.2%) (Table 1).

### 3.2. Participants’ Quality of Life

Due to the use of three different CFQ-R questionnaires, respondents’ quality of life scores are assessed and presented collectively for all patients and each age group separately in Table 2.

Amongst the whole group of patients, the eating problems domain scored the highest with a median of 88.89, followed by digestive symptoms (77.78) and physical functioning (75.00). The social functioning domain achieved the lowest median with 61.90 points. In every domain, at least one patient scored the highest possible score of 100 points; however, in emotional functioning and body image, at least one respondent scored the lowest with 0 points. The highest minimum score (33.33) was achieved in the digestive symptoms domain (Table 2).

As presented in Table 2, among the youngest participants (age 6–11), four of the eight domains achieved median scores of over 80 points, with the highest score in physical functioning (88.2). The lowest median was observed in social functioning (61.90), which also presented the lowest maximum score (71.43) amongst all of the domains. In the group of 12- and 13-year-olds, results were either the same (eating problems, treatment burden, social functioning, respiratory symptoms and digestive symptoms) or lower than in the younger group. The highest median was observed in eating problems and the lowest in social functioning (Table 2).

What is worth mentioning is that none of the participants younger than 14 years old assessed their perceived quality of life as the lowest possible (0.00) in any of the domains, whereas amongst patients in the 14+ group, some scored 0.00 in the vitality, emotional functioning, health perceptions, body image and weight domains. The oldest group surveyed assessed their quality of life as the best in the eating problems (88.89) domain and the lowest in vitality (50.00) (Table 2).

The data presented in Table 2 show clearly that in most domains quality of life is lower in the oldest group, with the most noticeable differences of 22.22 points lower in physical functioning and body image. High results, and the same medians (88.89) in every group, were found in the eating problems domain. Surprisingly, in the digestive symptoms domain the oldest group scored 16.67 points higher than others. With the exception of social functioning, where the difference was almost unnoticeable (1.98), it was the only domain where 14 years old and older patients scored higher than younger groups (Table 2).

### 3.3. Influence of the Quantitative Variables on Respondents’ Health-Related Quality of Life

The age of the participants significantly and negatively correlated with eight of the quality of life domains (*p* > 0.05). The strongest among the statistically significant correlations was observed with the age and treatment burden (r = −0.474), however, it was only fair correlation. Weight (rho = 0.447; r = 0.310), height (rho = 0.228; r = 0.225) and BMI (rho = 0.429; r = 0.257) were correlated with eating problems and digestive symptoms. BMI was positively and significantly correlated with all of the domains regarding nutrition and body. Saturation significantly correlated with physical functioning (rho = 0.405) and respiratory symptoms (rho = 0.324). Heart rate was not significantly correlated with any of the CFQ-R domains (Table 3).

### 3.4. Influence of the Spirometry Results on Respondents’ Health-Related Quality of Life

The physical functioning domain was positively correlated with all of the spirometry parameters; all the correlations were fair and significant. The strongest of those, and of all correlations, was observed with FEV1% (rho = 0.588). Treatment burden, body image and respiratory symptoms were also significantly positively correlated with all of the parameters except PEF%, however, almost all of the correlations were weaker than those with FEV1%. The only significant negative correlations were found between digestive symptoms and MEF% (rho = −0.225) and MEF75% (rho = −0.253), however, those were only poor. Emotional functioning, eating problems, health perceptions and social functioning were not significantly correlated with any of the parameters (Table 4).

### 3.5. Influence of the Qualitative Characteristics on Respondents’ Health-Related Quality of Life

The group of respondents with present exacerbations had significantly lower quality of life in 8 of 12 domains. The most affected was the health perception domain (Z = −3.045, *p* = 0.002) followed by weight (Z = −2.944, *p* = 0.003). The sex of the respondents did not influence their quality of life, however, those who were living in the rural areas presented higher scores in the domains of physical functioning (Z = 2.063, *p* = 0.039), body image (Z = 2.676, *p* = 0.007) and respiratory symptoms (Z = 0.2.236, *p* = 0.025). Patients surveyed during the COVID-19 pandemic presented higher scores for treatment burden (Z = 2.137, *p* = 0.033) and social functioning (Z = 2.102, *p* = 0.036) (Table 5).

#### 3.5.1. Multivariate and Univariate Analysis of Covariance for the Patients’ Health-Related Quality of Life

The MANCOVA model showed that the differences between health-related quality of life between patients according to their health condition were marginally significant (Wilks λ = 0.88, F = 2.42, and *p* = 0.057) after controlling for their FVC%, FEV1%, place of residence and for the COVID-19 pandemic (Table 6).

The univariate analysis of covariance (ANCOVA) confirmed the significant effect of the health condition on the social functioning domain (F = 8.32, *p* = 0.005), but not on the rest of the dependant variables. The COVID-19 pandemic this time had a significant effect on the social functioning domain (F = 5.89, *p* = 0.018). The place of residence had a significant effect on the body image domain (F = 5.60, *p* = 0.21) (Table 7).

#### 3.5.2. Health-Related Quality of Life of Patients, Depending on Their Health Condition

**Figure 1:**
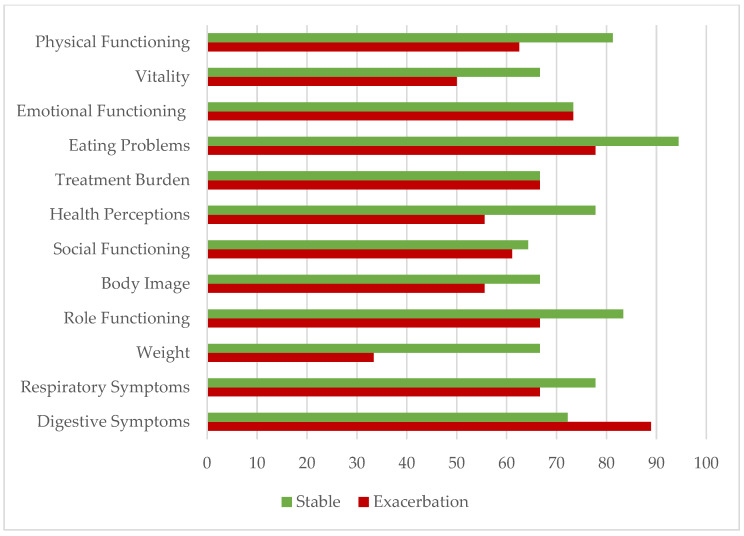
The comparison of health-related quality of life of those surveyed.

For a better understanding of the influence of exacerbations on respondents’ life, a comparison of the descriptive statistics was made for each of the quality of life domains and presented in Table 5. The simple comparison of the medians is presented in Figure 1. Patients with exacerbations had lower scores in almost all of the domains. Digestive symptoms were the only domain with the lower median in the stable group (72.23 vs. 88.89). The lowest median was observed in the weight domain, with the score being over two times lower in the exacerbations group (66.67 vs. 33.33). Weight was also the only domain with the lowest possible score in both groups, which is 0.00. Minimum scores were more diverse for both groups, whereas maximum scores were almost identical with 100.00 points. The only exception in the group with present exacerbations was vitality (83.33) (Table 8).

## 4. Discussion

The present study focuses on Polish CF patients’ health-related quality of life and factors that may influence it. While the issue is widely researched and described, there are not many studies that include this particular Polish population [18,23,24].

Among the younger group (6 to 13 years old), none of those surveyed scored the lowest possible score of 0 in any of the domains and their medians in the majority of the domains shared with patients 14 years old and older were higher. Participants assessed their quality of life the highest in the eating problems domain; all three groups had a median of 88.89 points, which shows that problems with eating are not very severe in the study group. According to other studies, the eating problems domain is one of the highest scoring domains, especially among adolescents and adults [23,25,26,27,28,29,30]. Physical functioning and respiratory symptoms also scored above 80 in the younger group, as did emotional functioning in the youngest group surveyed. Almost identical results were obtained in the study by Tepper et al. [31] in the group of 6- to 13-year-old patients. In a Polish study by Borawska-Kowalczyk and Sands [23], physical functioning was also the highest-scored domain in children aged 6 to 13 years old with 78 points, ex aequo with digestive symptoms. Interestingly, in our study, young participants scored rather low in the latter (66.67), while the oldest group had the second highest median (83.34) in that domain, which was similar to older patients in the Borawska-Kowalczyk and Sands study [23]. High scores in the aspects of digestion were repeated in other studies, suggesting that although there are aspects of CF that may cause digestive problems, patients do not find this aspect of their lives to be as negatively influenced by the disease as other aspects [24,29,32]. There were almost no differences between age groups and the assessment of their social functioning (61.90 vs. 61.91 vs. 63.89), which shows that all CF patients suffer in that part of their life regardless of their age. Social functioning was the lowest-scored domain in the study by Pattie et al. [33] conducted among children aged 6 to 10 years old. Vitality and body image were scored the lowest by the 14 years and older patients. Low vitality among adolescents and adults with CF seems to be a common phenomenon [23,26,27,28,31]. This seems to be a logical consequence of the disease that involves pulmonary and digestive problems. Problems with body image were the most pronounced in the study by Kir et al. [32]. When analysing the medians for that domain in our three research groups, we see that the scores for the body image domain decreased with age. Older patients tend to be more aware of the toll that their disease takes on their bodies and are more prone to compare their bodies to others. We found a comparable trend in treatment burden, where the median for this domain was lower by 10 points in the oldest group. Although it did not score the lowest in this group, it is common for treatment burden to be the lowest-scored domain by teenagers and adult CF patients [24,27,29,34]. The reason for this might be the natural progression of the disease, the different types of treatment that comes along with it and the time that it consumes. Children and younger teenagers assessed their quality of life as the lowest in terms of treatment burden in studies by Borawska-Kowalczyk and Sands [23] and Kir et al. [32]. Even though younger patients are usually healthier and require fewer treatments, it is understandable that for children the necessity of any treatments that take them away from playing or spending time with their peers can be a heavy burden. The lack of awareness of the severity of their illness and the emotional maturity that comes with age could be the reason for those results. It is important to take appropriate care of young CF patients so they can have the closest thing as possible to a normal childhood. Older teenagers and adults are more aware of the consequences of their disease and what life with it looks like. They rated their health perception rather low, showing that CF patients perceive their health as not being as good as that of others, which other studies confirm [18,23,27,30,31]. This was one of the most differentiated domains among those surveyed, and factors that may influence it should be examined in future studies.

The health-related quality of life of CF patients can be influenced by many factors [19]. In the present study, authors examined some of them, such as quantitative variables like body mass, BMI, age and lung function measured by spirometry, and qualitative variables such as patients’ sex, place of residence and the presence of exacerbations.

We found that the weight and BMI of those surveyed were significantly positively correlated with the eating problems and weight domains, which is not surprising, and is confirmed by other studies [19,26,28,29,35,36]. Patients with a higher BMI had significantly better body image, which is also similar to the results of previous studies [19,26,28,36]. However, patients from the Tomaszek et al. [37] research presented better body image with correlation to their weight. It is important to control patients’ nutrition state since eating disorders are common amongst people with CF, and malnutrition is associated with deteriorated quality of life [27,38,39]. Although insignificant, surveyed height was negatively correlated with seven of the domains, whereas weight and BMI with only four. Those results might be based on the difficulties in achieving proper weight gains by taller patients and it resulted in being skinnier and more prone to body image issues and chronic fatigue.

The age of the participants appeared to be an important factor in deteriorating quality of life. Age was negatively correlated with all of the domains, except digestive symptoms, indicating that with older age CF patients suffer less from digestive ailments. However, that correlation was statistically insignificant. The strongest correlations were found with treatment burden and physical functioning, which is not surprising considering the chronic nature of CF and deteriorating health with older age. Bodnar et al. [36] also found that older patients feel the burden of treatment more than younger ones. Similar conclusions were published in an article by Alishbayli et al. [40], where patients younger than 14 years old presented less treatment burden than older ones surveyed. Studies confirm the negative influence of age on general quality of life [19,21,35]. Younger age is associated with better quality of life, which confirms the study by Horck et al. [21], where the group of CF patients aged 6 to 18 years old was studied during the one-year follow-up. Amongst the majority of participants younger than 12 years old, quality of life had improved, whereas in the older patients it had deteriorated. Despite that, eating problems significantly improve with age in one of the studies [36], and, according to Alishbayli et al. [40], social functioning of patients 14 years and older is substantially higher than in younger groups surveyed.

Although spirometry is not the ideal tool and it does not give information about inflammation or pulmonary remodelling, and, according to some findings, in young children is not strongly correlated with their quality of life, it is still the standard examination performed on patients with CF in the facility where our study was conducted [11,33,41]. The 2-year study by Aquino et al. showed that routine spirometry significantly improved lung function and was associated with better identification of pulmonary exacerbations [42].

Amongst the spirometry measurements, FEV% is one of the most described and important determinants of CF patients’ quality of life. In the systematic review by Habib et al. [19], FEV% was positively correlated with all of the CFQ-R domains except for emotional functioning, social functioning and digestive symptoms. Our findings seem to confirm those results. FEV1% was positively correlated with every domain except digestive symptoms, but the correlations were statistically significant only for 6 out of 12 domains (physical functioning, treatment burden, body image, role functioning, weight and respiratory symptoms), with the strongest correlation being with physical functioning (0.588). The positive correlation of the physical functioning domain with FEV1% is well documented in many research papers [26,29,35,36,37,40,43,44]. Similar to our results, FEV1% positively correlated with treatment burden in studies by Santana et al. [45], Gancz et al. [29] and Quittner et al. [35]. Body image and weight were also correlated with FEV1% in that study and others [28,36,37]. A study by Bodnar et al. [36], similar to ours, found a negative correlation between FEV1% and the digestive symptoms domain. Similar results were found in a paper by Quittner et al. [35].

Forced vital capacity is a less studied determinant of health-related quality of life among people with CF. Our study showed that FVC% correlations were almost identical to those with FEV1%. FVC% was positively and significantly correlated with nine domains, with the only difference coming from FEV1% in vitality. Santana et al. [45] found that only social functioning was significantly correlated with FEV, which is contradictory to our findings. Also, results from Moço et al. [25] did not align with ours. They found that treatment burden and digestive symptoms were negatively correlated with FVC% [25].

In our study, male patients scored higher in every domain, except weight, however, those differences were not statistically significant. Considering the lack of statistical significance of our results, we conclude that in our study group the sex of the participants was not associated with their perceived quality of life. Some studies align with our results [29,34,36,40]. Analysing the literature, a trend of the male gender as a determinant of better general quality of life was found [27,37,45,46]. Physical functioning was significantly higher in male CF patients in the systematic review by Habib et al. [19], and in the studies by Ancel et al. [28] and Tomaszek et al. [37]. Borawska and Sands [23] found that Polish boys aged 14 to 18 years old with CF reported a significantly higher quality of life in the physical functioning domain. In the Swedish study by Hochwälder et al. [27], adult males, in comparison to their female peers, presented higher quality of life in eight out of nine domains raw, but the differences were insignificant. What is worth noticing is that the only significant differences were found in the weight and body image domains, with females scoring higher. This observation seems to be repeated in other studies. Ancel et al. [28] found that amongst CF patients, male sex was a significant predictor of lower weight scores. Similarly, amongst the patients aged 12 years and older in a study by Hebestreit et al. [26], the median of the scores in the weight domain for females was 100 and for males 66.7. Those results might be the reflection of body image in today’s society, where women are expected to have slimmer bodies in comparison to men, who are expected to be able to build muscles and have stronger bodies.

The place of residence can be related to patient’s quality of life. We found that those surveyed who were living in villages had significantly higher quality of life in terms of the physical functioning, body image and respiratory symptoms domains in comparison to those who were living in small and big cities. The results for body image were confirmed by the univariate analysis of the MANCOVA model, where place of residence had a significant effect on that domain. While big cities offer more opportunities for healthcare services, which potentially should improve CF residents’ health, smaller towns and rural areas usually are related to a calmer, more balanced lifestyle and a healthier environment. All of that could have contributed to better physical functioning, body image and fewer respiratory symptoms in our study sample. In the Turkish study, demographic variables were not associated with quality of life [34].

Cystic fibrosis is a lifelong disease associated with the occurrence of numerous exacerbations often requiring hospitalisations and an intravenous administration of antibiotics [47,48]. Due to that reason, our study survey was also administered across two groups: those with present exacerbations and those who were stable. During the statistical analysis we found that patients with exacerbations had lower scores in every domain, except the digestive symptoms domain, but not all the differences were significant. A significant difference was found in 8 out of 12 domains, with the highest difference in the health perceptions (Z = −3.045; *p* = 0.002), weight (Z = −2.944; *p* = 0.003) and role functioning (Z = −2.031; *p* = 0.042) domains, showing that exacerbations are an important factor negatively influencing patients’ quality of life. This could indicate that during exacerbations patients’ perception of health decreases, they have difficulties in gaining weight and are not able to properly maintain their social roles. This is not unexpected because the decline in health of people with CF often results in the need for hospitalisation and intensified pharmacological and physiotherapeutic treatments. We did not analyse the type of exacerbations, so we cannot tell if they were pulmonary, digestive or infectious reasons, however, all our patients from the exacerbation group were hospitalised. So, it is not unexpected for patients being hospitalised to assess poorly their health, vitality and physical aspect of functioning or to report having more respiratory symptoms. All of the previously mentioned aspects and the hospitalisation can contribute to deteriorations in social functioning and in fulfilling their social roles. We also confirmed the influence of exacerbations on patients’ social functioning by the univariate analysis of MANCOVA model, confirming their significant impact on the quality of life of surveyed patients. Exacerbations are the cause of deterioration in the quality of life in other studies [39,44,48,49]. In the retrospective study by Waters et al. [47], almost half of those surveyed had at least one pulmonary exacerbation per year, and those occurring more frequently than every 6 months were associated with a decline in lung function. The hospitalisations in the previous 12 months were associated with lower physical functioning in studies by Alishbayli et al. [40] and Bodnar et al. [36]. In the patients studied by Flume et al. [49], physical functioning and vitality were the domains that took the longest to return to the state from before the hospitalisation due to exacerbations. Despite that, in our study sample, hospitalised patients did not rate their burden of treatment as being significantly lower than those in a stable condition, and some studies show that it is correlated and that the treatment burden domain score decreases with the complexity of treatment and the degree of exacerbation [21,34]. Although antibiotic treatment is not free from its side effects, it was correlated with increased scores in the respiratory symptoms domain and decreased bacterial density and sputum inflammatory markers after pulmonary exacerbations in a study by Hoppe et al. [50].

Due to the COVID-19 pandemic and the lockdown ordered in Poland during the study period, the time of the data collection was additionally incorporated into the study as a categorical variable as before and during the pandemic. Although, while comparing those two groups, we achieved significant differences in treatment burden and social functioning domains, the multivariate analysis did not confirm those results. However, the univariate analysis found there to be a significant effect of the COVID-19 pandemic on the social functioning domain. Those findings suggest that CF patients coped better with their treatment regime and had a better social life during the COVID-19 pandemic and lockdown. Though those results might be surprising, it is important to remember what the everyday life of people living with CF looks like. Home isolation, strict treatment schedule, numerous exacerbations often leading to hospitalisations are nothing new for them. Lockdown forced many telehealth solutions that allowed online consultation and at-home monitoring, leading to fewer hospital visits, which could have reduced the burden of their treatment. The necessity of transferring social interactions to online forms may have made it easier for people with CF to establish and maintain social contacts and relationships. Experiences gathered during the pandemic seems to confirm that patients were not negatively influenced by the pandemic and first lockdowns in terms of their treatments and social lives. According to Boni et al., Italian people with CF during the pandemic were more likely to follow daily respiratory physiotherapy despite their decrease in physical activity [51]. In Hatziagorou et al.’s study, the majority of patients not only did not reduce their daily exercise frequency, but also increased their body weight and FEV1 [52]. Some studies show that financial status might have influenced the physical activity of CF patients during the pandemic, since those with better financial status were more active and maintained social contact with their friends and families [53]. Telehealth rapidly evolved during the pandemic, and although it needs further improvements and research it seems to be an effective and convenient new tool in CF care [54,55,56,57,58].

The study has some limitations. Since the data collection was partially performed during the COVID-19 pandemic and its few waves in Poland, the study sample is small, and the age groups are not even. As a result of the very limited numbers of participants in the groups with children of age 6 to 11 and 12 to 13 years old, their results had to be summed with the rest of the participants for the statistical analysis. Nevertheless, it should be remembered that CF is a rare disease. In 2018, in Podkarpackie Voivodeship where the study was conducted, only 92 patients used the healthcare service financed by the National Health Fund [4]. Also, the research was conducted in only one of the Polish Cystic Fibrosis Centres, thus, the study group may not accurately represent the entire population of CF patients living in Poland. The wide age range of participants may be another limitation of the study. Multi-centred nationwide research is needed to collect data in appropriately large age groups or focused on a specific age group in order to obtain results that reflect well the situation of patients throughout the country and in specific age groups. Another limitation of the study is the fact of not analysing the types, causes and severity of present exacerbations among study participants. Inclusion of those characteristics would be beneficial for understanding the role of different type of exacerbations on CF patients’ quality of life. It is a potential new research area that authors will study in their future research.

## 5. Conclusions

In the present study, we assessed CF patients’ health-related quality of life. Respondents from the youngest groups presented with higher quality of life in almost all of the CFQ-R domains. Patients aged 14 years old and older suffered from low vitality. When analysing obtained scores from each group, altogether the highest HRQoL score was in eating problems, and the lowest in social functioning, treatment burden and body image. Our results indicate that it is important to focus attention on those aspects of patients’ lives and provide them with the necessary support from their healthcare providers.

Age and exacerbations were factors negatively influencing CF patients’ health-related quality of life. Due to the introduction of new medications such as CFTR modulators as one of the treatment options, it is possible that the life expectancy of Polish CF patients will be extended. However, we do not know yet what quality of life those additional years would have, and if they would be free from exacerbations and other complications. It may be a new challenge for all healthcare professionals to provide care for ageing CF patients, and to deal with possible decreasing quality of life in those later years.

The COVID-19 pandemic positively influenced the social functioning of CF patients. Also, those that lived in villages had better images of their bodies. Such findings might suggest that the place of living plays an important role in satisfaction with their own bodies, and that not only are CF patients well adapted to social isolation, but were also able to maintain social relationships during the period of lockdown.

High percentage predicted values of all of the spirometry results were associated with better physical functioning, but FVC%, FEV1% and MEF25% were the parameters that positively correlated with the highest number of CFQ-R domains, indicating that declines in those parameters might be a predictor of deteriorations in patients’ quality of life. CF healthcare providers should monitor those parameters during patients’ routine spirometry since it can provide them with valuable information about patients’ quality of life.

## Figures and Tables

**Table 1:** Baseline characteristics of the participants.

Characteristic		n	%
Sex	Female	47	59.5
	Male	32	40.5
Gender distribution of participants	Boys *	13	16.5
	Men **	19	24.0
	Girls *	12	15.2
	Women **	35	44.3
Age	6–11	10	12.7
	12–13	7	8.9
	14–42	62	78.5
Place of residence	Village	55	69.6
	City under 100,000 citizens	18	22.8
	City over 100,000 citizens	6	7.6
Educational level	In high school	38	48.1
	High school	31	39.2
	Vocational education	4	5.1
	Higher education	6	7.6
Marital status **	Single	44	81.5
	Married	6	11.1
	Divorced	4	7.4
Health condition	Stable	32	40.5
	Exacerbations	47	59.5
COVID-19 pandemic	Before the pandemic	37	46.8
	During the pandemic	42	53.2

* Variable includes only underage participants—up to 17 years old; ** characteristic calculated only for the adult participants; n: number of participants; and %: percentage.

**Table 2:** Health-related quality of life of respondents aged 6 to 11 (n = 9), 12 to 13 (n = 8), 14 years old or older (n = 62) and all of the participants (n = 79).

HRQoL Domain	Age Group	Mean	Median	Interquartile Range	Minimum	Maximum
Physical Functioning	6–11	88.27	88.89	16.67	72.22	100.00
	12–13	81.25	83.33	19.44	66.67	100.00
	14+	63.17	66.67	45.83	12.50	100.00
	All	67.86	75.00	45.84	12.50	100.00
Vitality *	14+	53.63	50.00	25.00	0.00	100.00
Emotional Functioning	6–11	79.17	83.33	16.67	66.67	87.50
	12–13	77.08	77.09	18.75	62.50	87.50
	14+	67.20	73.33	26.67	0.00	100.00
	All	69.57	73.33	23.33	0.00	100.00
Eating Problems	6–11	75.31	88.89	33.33	44.44	100.00
	12–13	83.34	88.89	22.22	55.56	100.00
	14+	79.93	88.89	33.33	11.11	100.00
	All	79.75	88.89	33.33	11.11	100.00
Treatment Burden	6–11	75.31	77.78	11.11	55.56	88.89
	12–13	76.39	77.78	11.11	44.44	100.00
	14+	63.08	66.67	11.11	22.22	100.00
	All	65.82	66.67	22.22	22.22	100.00
Health Perceptions *	14+	56.81	66.67	44.45	0.00	100.00
Social Functioning	6–11	60.32	61.90	4.76	47.62	71.43
	12–13	61.91	61.91	28.57	38.10	85.71
	14+	63.71	63.89	22.22	22.22	100.00
	All	63.14	61.90	22.22	22.22	100.00
Body Image	6–11	77.78	77.78	22.22	55.56	100.00
	12–13	75.00	66.67	22.22	66.67	88.89
	14+	58.24	55.56	44.45	0.00	100.00
	All	62.17	66.67	33.34	0.00	100.00
Role Functioning *	14+	72.85	75.00	25.00	33.33	100.00
Weight *	14+	55.91	66.67	66.67	0.00	100.00
Respiratory Symptoms	6–11	81.48	83.33	8.33	66.67	100.00
	12–13	79.17	83.33	25.01	50.00	100.00
	14+	66.67	66.67	22.22	16.67	100.00
	All	69.62	72.22	22.22	16.67	100.00
Digestive Symptoms	6–11	62.04	66.67	33.34	33.33	100.00
	12–13	75.00	66.67	23.57	33.33	100.00
	14+	82.44	83.34	33.33	44.44	100.00
	All	79.36	77.78	33.33	33.33	100.00

* Health-related quality of life domains occurring only in the CFQ-R 14+ questionnaire and HRQoL: health-related quality of life.

**Table 3:** Health-related quality of life domain’s scores and patients’ qualitative characteristics correlations.

HRQoL Domain	Weight [kg]	Height [cm]	BMI	Saturation	Heart Rate	Age
Physical Functioning	−0.065	−0.213	0.085	0.405 **	−0.156	−0.428 **
Vitality *	0.053	−0.063	0.048	0.140	0.062	−0.319 **
Emotional Functioning	−0.040	−0.143	−0.075	0.016	0.095	−0.113
Eating Problems	0.447 **	0.228 **	0.429 **	0.143	−0.042	−0.068
Treatment Burden	−0.173	−0.134	−0.166	0.140	−0.062	−0.474 **
Health Perceptions *	0.080	0.035	−0.031	0.104	0.125	−0.296 **
Social Functioning	0.128	0.090	0.080	−0.026	0.078	−0.114
Body Image	0.191	−0.101	0.394 **	0.096	−0.066	−0.407 **
Role Functioning *	0.229	0.095	0.127	0.165	−0.031	−0.261 **
Weight *	0.358 **	−0.112	0.537 **	0.092	−0.030	−0.280 **
Respiratory Symptoms	−0.045	−0.103	−0.038	0.324 **	−0.061	−0.447 **
Digestive Symptoms	0.310 **	0.225 **	0.257 **	−0.232 **	0.155	0.214

* Health-related quality of life domains occurring only in the CFQ-R 14+ questionnaire; ** *p* < 0.05; HRQoL: health-related quality of life; and BMI: body mass index.

**Table 4:** Health-related quality of life domain’s scores and patients’ spirometry results correlations.

HRQoL Domain	VC%	FVC%	FEV1%	FEV1/FVC%	PEF%	MMEF%	MEF 25%	MEF 50%	MEF 75%
Physical Functioning	0.532 **	0.569 **	0.588 **	0.453 **	0.454 **	0.512 **	0.549 **	0.501 **	0.476 **
Vitality *	0.253 **	0.269 **	0.229	0.130	0.022	0.146	0.228	0.212	0.043
Emotional Functioning	0.062	0.055	0.055	0.043	−0.064	−0.028	0.039	0.037	−0.041
Eating Problems	0.149	0.173	0.141	−0.004	0.063	0.055	0.048	0.034	0.023
Treatment Burden	0.229 **	0.285 **	0.280 **	0.227 **	0.148	0.235 **	0.235 **	0.243 **	0.264 **
Health Perceptions *	0.183	0.190	0.166	0.060	0.007	0.083	0.138	0.120	0.027
Social Functioning	0.024	0.001	0.048	0.035	−0.027	0.010	0.039	0.019	−0.035
Body Image	0.242 **	0.320 **	0.346 **	0.254 **	0.167	0.260 **	0.331 **	0.284 **	0.236 **
Role Functioning *	0.219	0.238	0.265 **	0.227	0.048	0.208	0.251 **	0.259 **	0.165
Weight *	0.104	0.251 **	0.266 **	0.200	0.106	0.204	0.254 **	0.234	0.169
Respiratory Symptoms	0.338 **	0.368 **	0.371 **	0.330 **	0.185	0.376 **	0.399 **	0.394 **	0.291 **
Digestive Symptoms	−0.112	−0.140	−0.158	−0.171	−0.179	−0.225 **	−0.142	−0.208	−0.253 **

* Health-related quality of life domains occurring only in the CFQ-R 14+ questionnaire; ** *p* < 0.05.

**Table 5:** Comparison of patients’ health-related quality of life according to their qualitative characteristics.

HRQoL Domain	Male Sex		Village as the Place of Residence		Present Exacerbations		COVID-19 Pandemic	
	Z	*p* Value	Z	*p* Value	Z	*p* Value	Z	*p* Value
Physical Functioning	0.145	0.885	2.063	0.039 **	−2.754	0.006 **	−0.192	0.848
Vitality *	1.787	0.074	1.347	0.178	−2.764	0.006 **	−0.556	0.578
Emotional Functioning	0.201	0.841	0.568	0.570	−0.898	0.369	1.046	0.295
Eating Problems	0.072	0.943	−0.132	0.895	−1.686	0.092	−0.948	0.343
Treatment Burden	1.343	0.179	1.549	0.121	−1.649	0.099	2.137	0.033 **
Health Perceptions *	0.989	0.323	1.241	0.214	−3.045	0.002 **	0.387	0.699
Social Functioning	0.401	0.688	0.289	0.773	−2.150	0.032 **	2.102	0.036 **
Body Image	0.580	0.562	2.676	0.007 **	−2.446	0.014 **	0.422	0.673
Role Functioning *	1.427	0.154	1.623	0.105	−2.031	0.042 **	0.542	0.588
Weight *	−0.603	0.546	0.132	0.895	−2.944	0.003 **	−1.161	0.245
Respiratory Symptoms	1.925	0.054	2.236	0.025 **	−2.336	0.020 **	−0.108	0.914
Digestive Symptoms	0.738	0.460	0.512	0.608	1.224	0.221	1.179	0.238

* Health-related quality of life domains occurring only in the CFQ-R 14+ questionnaire; ** *p* < 0.05; and HRQoL: health-related quality of life.

**Table 6:** The MANCOVA model results for association between the patients’ health condition, place of residence, COVID-19 pandemic, FVC%, FEV1% and health-related quality of life domains: physical functioning, social functioning, body image and respiratory symptoms.

Model	Wilks λ	F	*p* Value
Intercept	0.29	42.75	0.000 *
FVC%	0.94	1.12	0.354
FEV1%	0.98	0.38	0.825
Health condition (stable–exacerbations)	0.88	2.42	0.057 **
Place of residence (village–city)	0.91	1.78	0.142
COVID-19 (before pandemic–during pandemic)	0.88	2.36	0.061

* *p* < 0.05; ** 0.05 < *p* < 0.06.

**Table 7:** Results of univariate analysis of MANCOVA model for health-related quality of life domains: physical functioning, social functioning, body image and respiratory symptoms.

Model		F	*p* Value
Physical Functioning	Intercept	5.36	0.023
	FVC%	0.94	0.336
	FEV1%	1.11	0.296
	Health condition (stable–exacerbations)	0.41	0.523
	Place of residence (village–city)	0.81	0.371
	COVID-19 (before pandemic–during pandemic)	0.41	0.524
Social Functioning	Intercept	155.10	0.000
	FVC%	0.78	0.381
	FEV1%	0.04	0.842
	Health condition (stable–exacerbations)	8.32	0.005 *
	Place of residence (village–city)	0.37	0.546
	COVID-19 (before pandemic–during pandemic)	5.89	0.018 *
Body Image	Intercept	34.30	0.000 *
	FVC%	0.45	0.506
	FEV1%	0.02	0.901
	Health condition (stable–exacerbations)	3.22	0.077
	Place of residence (village–city)	5.60	0.021 *
	COVID-19 (before pandemic–during pandemic)	0.05	0.823
Respiratory Symptoms	Intercept	23.03	0.000 *
	FVC%	1.28	0.262
	FEV1%	0.07	0.799
	Health condition (stable–exacerbations)	1.31	0.257
	Place of residence (village–city)	3.47	0.066
	COVID-19 (before pandemic–during pandemic)	0.09	0.764

* *p* < 0.05.

**Table 8:** Comparison of health-related quality of life of stable patients and those with present exacerbations.

HRQoL Domain	Median		Interquartile Range		Minimum		Maximum	
	Stable	EX	Stable	EX	Stable	EX	Stable	EX
Physical Functioning	81.25	62.50	26.39	41.66	25.00	12.50	100.00	100.00
Vitality *	66.67	50.00	33.33	33.34	25.00	0.00	100.00	83.33
Emotional Functioning	73.33	73.33	25.42	26.67	40.00	0.00	100.00	100.00
Eating Problems	94.45	77.78	33.33	33.33	44.44	11.11	100.00	100.00
Treatment Burden	66.67	66.67	11.11	22.22	44.44	22.22	100.00	100.00
Health Perceptions *	77.78	55.56	33.34	33.34	22.22	0.00	100.00	100.00
Social Functioning	64.29	61.11	26.19	16.67	38.10	22.22	100.00	100.00
Body Image	66.67	55.56	33.33	44.45	22.22	0.00	100.00	100.00
Role Functioning *	83.33	66.67	25.00	25.00	41.67	33.33	100.00	100.00
Weight *	66.67	33.33	33.33	33.34	0.00	0.00	100.00	100.00
Respiratory Symptoms	77.78	66.67	27.78	22.22	16.67	22.22	100.00	100.00
Digestive Symptoms	72.23	88.89	23.61	33.33	33.33	33.33	100.00	100.00

* Health-related quality of life domains occurring only in the CFQ-R 14+ questionnaire; HRQoL: Health-related quality of life; and EX: exacerbation.

## Data Availability

Data available on request due to restrictions privacy.
